# Case Report: Interest of Positron Emission Tomography in Pediatric Small Vessel Primary Angiitis of the Central Nervous System

**DOI:** 10.3389/fped.2022.794294

**Published:** 2022-03-07

**Authors:** Morgane Belcour, Pauline Dontaine, Anne Monier, Laetitia Lebrun, Isabelle Salmon, Olivier De Witte, Serge Goldman, Xavier De Tiège, Alec Aeby

**Affiliations:** ^1^Department of Pediatrics, Hôpital des Enfants Reine Fabiola (HUDERF), Université Libre de Bruxelles (ULB), Brussels, Belgium; ^2^Department of Pediatric Neurology, Université Libre de Bruxelles - Hôpital Universitaire Des Enfants Reine Fabiola (ULB-HUDERF), Brussels, Belgium; ^3^Department of Anatomopathology, Cliniques Universitaires de Bruxelles (CUB) Hôpital Erasme, Université Libre de Bruxelles (ULB), Brussels, Belgium; ^4^Department of Neurosurgery, Cliniques Universitaires de Bruxelles (CUB) Hôpital Erasme, Université Libre de Bruxelles (ULB), Brussels, Belgium; ^5^Department of Functional Neuroimaging, Service of Nuclear Medicine, Cliniques Universitaires de Bruxelles (CUB) Hôpital Erasme, Université Libre de Bruxelles (ULB), Brussels, Belgium

**Keywords:** primary angiitis of central nervous system, positron emission tomography (PET), cerebral vasculitis, magnetic resonance imaging (MRI), brain biopsy

## Abstract

Primary angiitis of the central nervous system (PACNS) is a rare inflammatory disease affecting central nervous system vessels. The diagnosis, which requires confirmation by brain biopsy, remains challenging due to unspecific clinical presentation and low specificity of imaging and laboratory exams. In these two pediatric biopsy-proven cases of svPACNS we demonstrate that brain positron emission tomography (PET) show a high metabolic activity that extends beyond brain MRI abnormalities. Therefore, combining MRI and PET abnormalities to adequately guide brain biopsy might increase the diagnostic yield of this rare condition.

## Introduction

Primary angiitis of the central nervous system (PACNS) is an inflammatory brain disease affecting the central nervous system (CNS) vessels (1). The clinical presentation is determined by the affected vessel size. At onset, large and medium artery disease present with acute ischemic or hemorrhagic stroke (2). Small vessel PACNS (svPACNS) is characterized by a chronic presentation with headaches (50–100% according to the cohorts), focal neurological deficits (37–75%), encephalopathy (17–100%), and seizures (50–100%) (2). Blood markers of systemic inflammation are usually normal or slightly elevated, and cerebrospinal fluid (CSF) analysis may show unspecific lymphocytic pleocytosis and elevated protein levels (1). Magnetic resonance imaging (MRI) changes concern 90–100% of the patients with svPACNS and include unspecific tumor-like lesions, leptomeningeal or parenchymal lesions with gadolinium enhancement, cortical and subcortical T2 or fluid attenuated inversion recovery (FLAIR) hyperintensities (3). Angiography has a low sensitivity (20–30%) and specificity (30%), and is usually negative in svPACNS (3). Differential diagnosis is wide and many pathologies can mimic svPACNS (2). Because of the low sensitivity and specificity of the clinical presentation, imaging and laboratory examinations, brain biopsy remains the gold standard to secure the diagnosis before heavy immunosuppressive drug therapy. Nevertheless, brain biopsy may be inconclusive because of the frequent sampling error due to the segmental nature of svPACNS (4). Supplementary diagnostic tools are therefore highly desirable when svPACNS is suspected.

Brain positron emission tomography (PET) seems an interesting imaging modality that could theoretically point to brain region(s) with active inflammation (3) and therefore be a useful tool in the diagnostic workup of this rare disease. Moreover, because of the segmental nature of svPACNS and the risk of sampling error, it could also improve the spatial targeting of brain biopsy as demonstrated for brain tumors (5). Finally, as many pathologies can mimic svPACNS, PET might also be useful to exclude extra-CNS inflammation.

Therefore, the aim of this case study is to report two biopsy-proven pediatric cases of svPACNS that demonstrate the utility of [^18^F]–fluorodeoxyglucose (FDG) and [^11^C]-methionine (MET) PET in the management and diagnosis of this rare condition.

## Case Report

**Figure 1:**
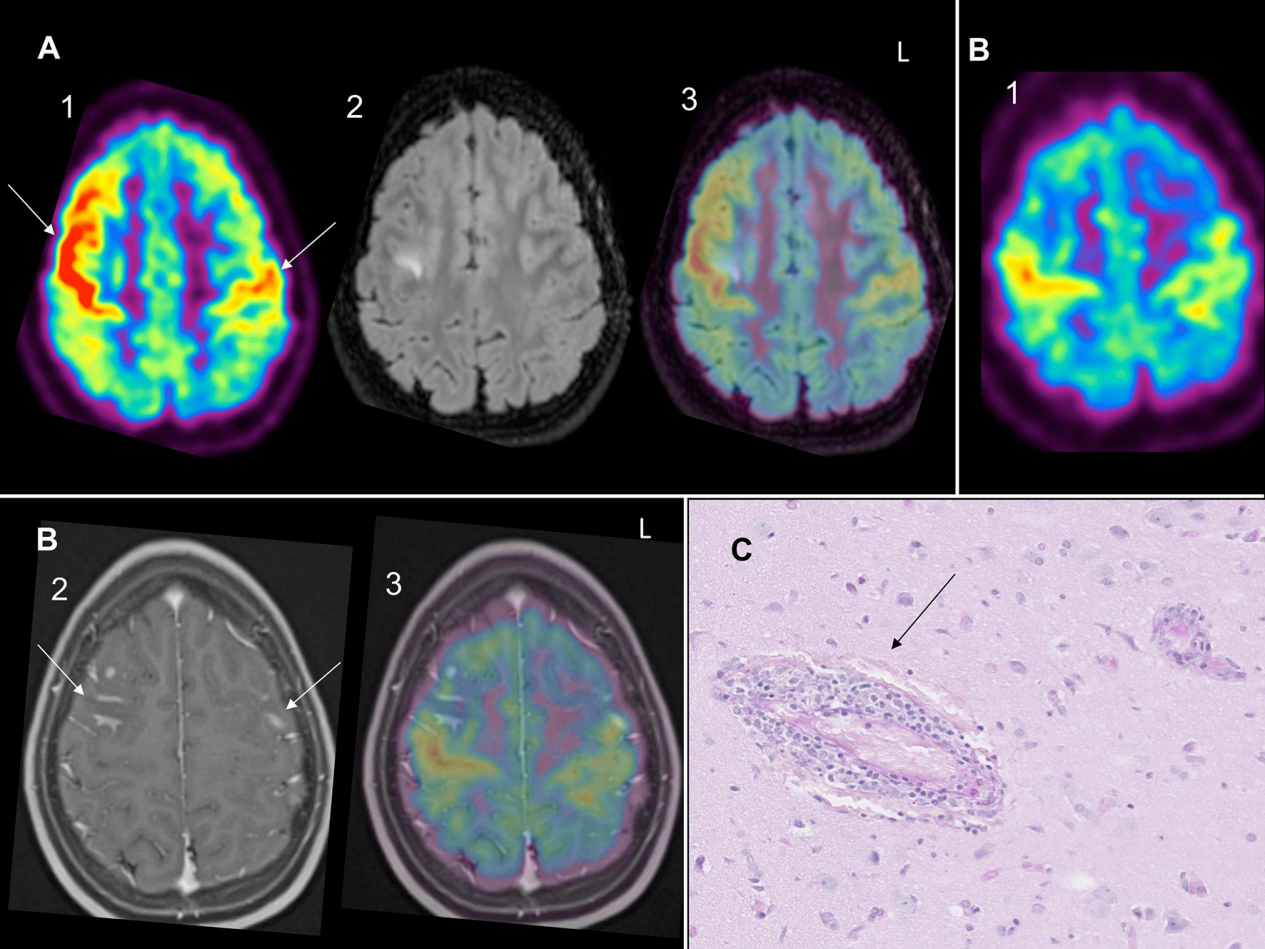
**(A)** (1) Bilateral right> left (arrows) PET-FDG increased uptake in both frontal lobe cortices with (2) right FLAIR hypersignal and (3) PET-MR coregistration showing mismatch between PET hypermetabolism and FLAIR hypersignal. **(B)** (1) Bilateral right>left PET-FDG increased uptake in both frontal lobes with (2) bilateral (arrows) enhancement on T1 gadolinium and (3) PET-MR coregistration showing mismatch between PET hypermetabolism and T1 gadolinium enhancement. **(C)** Small vessels non-granulomatous lymphocytic svPACNS on Periodic Acid Schiff (PAS) coloration on histology (arrow).

**Figure 2:**
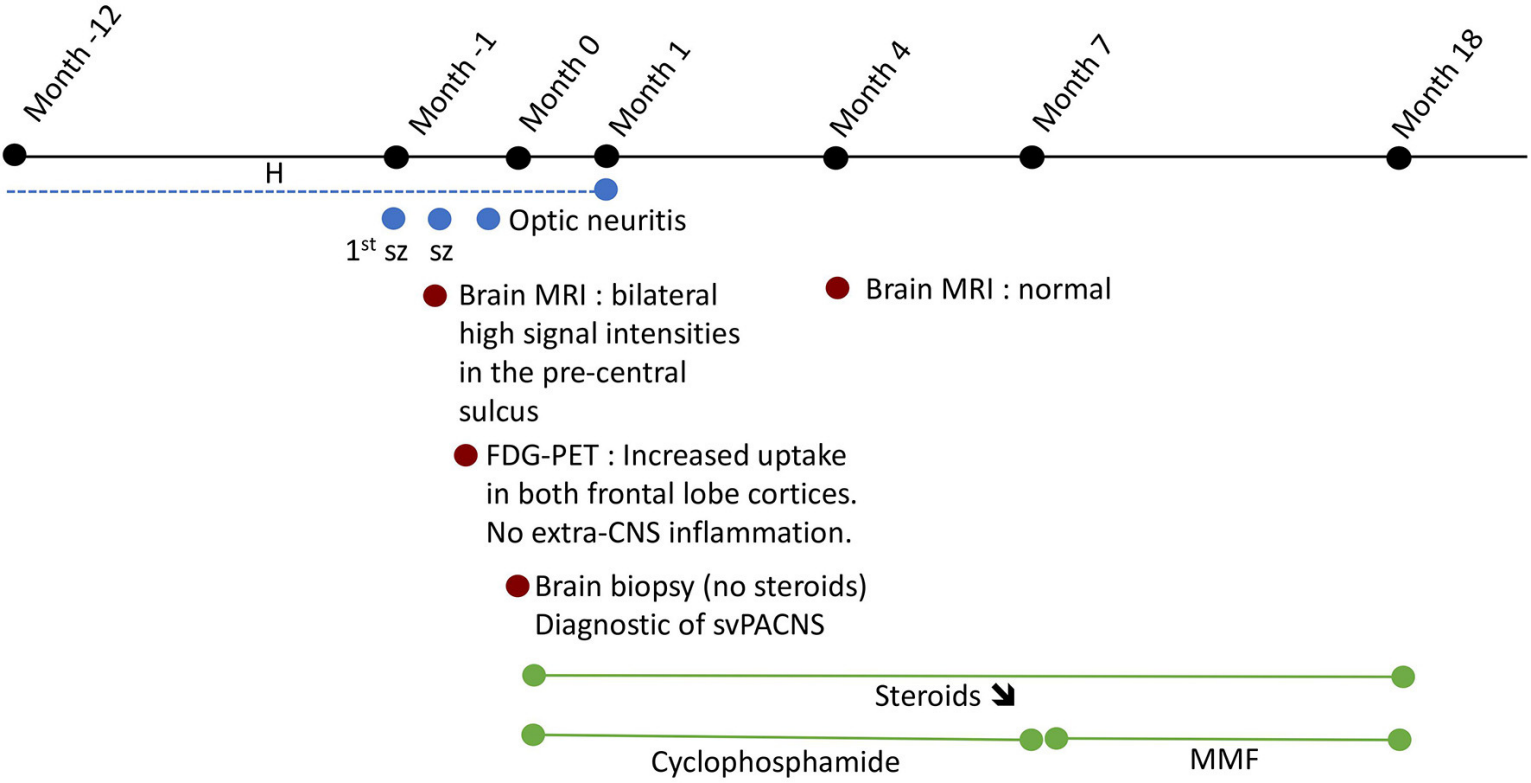
Time 0 represents the time of diagnostic of svPACNS. The dotted lines represent the intermittent nature of the complaints. H, headaches; MMF, mycophenolate mofetil; sz, seizure; **↘**, progressive weaning.

Patient 1 was a previously healthy 12-year-old girl, who presented an episode of loss of consciousness followed by transitory aphasia and paresthesia of the right upper limb suggestive of a focal seizure. In the emergency room, physical and neurological examination were unremarkable. The initial blood test and toxicological analysis were inconclusive. Conventional electroencephalogram (EEG) was normal and she was discharged with the scheduling of a brain MRI. Two days later, she presented a second episode associated with tonico-clonic movements of the right upper limb, deviation of the head and gaze to the right. On second admission, she also complained of chronic fatigue and recurrent headaches for 1 year. Blood tests showed an elevated sedimentation rate at 26 mm/h. The CSF revealed pleocytosis (16 leukocytes per μL, 80% lymphocytes). The extensive autoantibody search was negative, especially for anti-nuclear antibody (ANA), antineutrophil cytoplasmic antibody (ANCA), rheumatoid factor (RF) and anti-myelin oligodendrocyte glycoprotein (MOG). Brain MRI showed high signal intensities on T2-weighted imaging (-WI) and FLAIR images in the precentral sulcus bilaterally (Figure 1A) with enhancement on the T1-WI post-contrast images (Figure 1B) and vasogenic edema on diffusion-weighted images. EEG showed bilateral right>left frontal spike-waves. Whole body FDG-PET showed an increased right>left FDG-uptake in both frontal lobes (Figures 1A,B) and no extra CNS inflammation. PET-MR coregistration showed a mismatch between MRI and PET abnormalities (Figures 1A,B). We suspected svPACNS and we scheduled a brain biopsy to confirm the diagnosis. A few days later, she developed right eye pain, blurred vision and photophobia. The ophthalmological examination, which was normal on admission, showed bilateral papillary edema compatible with optic neuritis. This prompted us to perform a brain biopsy guided by MRI and FDG-PET with sampling of both MRI and PET abnormalities. The biopsy showed a lymphocytic predominant svPACNS (Figure 1C). She was treated with intravenous methylprednisolone pulse 30 mg/kg/d for 5 days followed by oral prednisone 2 mg/kg/d associated with levetiracetam 50 mg/kg/d and aspirin 5 mg/kg/d. Induction therapy also included a monthly dose of cyclophosphamide (600 mg/m^2^) for 7 months. Her symptoms progressively resolved and the antiepileptic treatment could be stopped. The ophthalmological examination was normal after 1 month of treatment, as well as the brain MRI in the middle of this first phase of treatment. The maintenance therapy included oral prednisone that was monthly tapered and mycophenolate mofetil daily (2 g/day) for one more year. The treatment was well-tolerated and she is currently in remission for 24 months under this regimen. A timeline with relevant data from the episode of care is represented in Figure 2.

**Figure 3:**
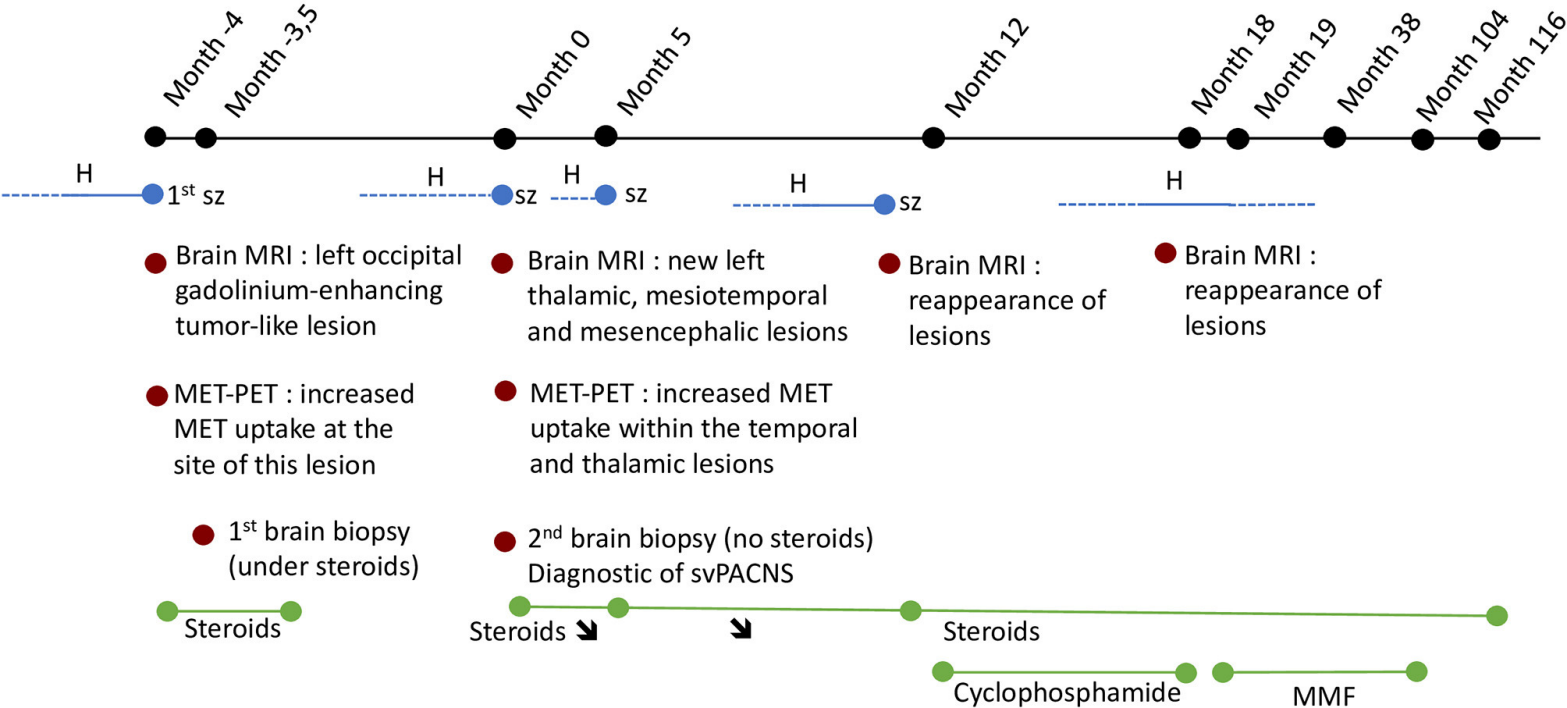
Time 0 represents the time of diagnostic of svPACNS. The dotted lines represent the intermittent nature of the complaints. H, headaches; MMF, mycophenolate mofetil; sz, seizure; **↘**, progressive weaning.

Patient 2 has already been reported elsewhere (6). A 9-year-old boy with progressive headaches presented focal to bilateral tonic-clonic seizures. Brain MRI showed a left occipital gadolinium-enhancing tumor-like lesion. Brain PET evidenced an increase in MET uptake at the site of this lesion, see Figure 1A in (6). Stereotactic brain biopsy performed under MRI and MET-PET guidance 12 days after the initiation of valproate and steroids revealed non-specific ischemic neurons and gliosis. Two months after presentation and weaning from steroids, brain MRI showed resolution of the occipital lesion but detected new gadolinium-enhancing left mesiotemporal, midbrain, and thalamic lesions. CSF analyses revealed pleocytosis (40 leukocytes per μL, 91% lymphocytes) and hyperproteinorrachia (50 mg/dL). Whole-body FDG-PET combined with computed tomography revealed increased glucose metabolism in the cervical and thoracic spinal cord and no extra-CNS inflammation. Within 2 months, he developed recurrent headaches, acute photophobia, emesis, and incoherent speech with an increase in the size of the left-brain lesions with edema and mass effect. PET revealed increased MET uptake within the temporal and thalamic lesions with a mismatch between FLAIR hypersignal and PET-MET uptake in several brain regions, see Figure 1C in (6). Stereotactic temporal brain biopsy under MRI and MET-PET guidance revealed svPACNS, see Figure 2 in (6). Methylprednisolone led to the resolution of the clinical symptoms and MRI abnormalities. Thirty-eight months after initial presentation, he became clinically and radiologically stable, under a combination of steroids and mycophenolate mofetil. He could stop all immunosuppressive and steroid sparing agents after 7 years and he is asymptomatic since then after a 5-year period. A timeline with relevant data from the episode of care is represented in Figure 3.

## Discussion

These two biopsy-proven cases of svPACNS demonstrate (i) that whole-body FDG-PET can contribute to exclude extra-CNS involvement, (ii) that FDG- and MET-PET are valuable additional diagnostic tools and should be performed in every suspected case of svPACNS, and (iii) that brain FDG and MET-PET can be complementary to brain MRI for the guidance of brain biopsy, the gold standard for the diagnosis of this rare neurological condition.

FDG-PET is based on the introduction of a positron emitter on a glucose analog that is administered intravenously (7). The earliest changes in inflammation are tissue hyperemia, enhanced vascular permeability, release of inflammatory mediators, and increased tissue blood perfusion (7). As inflammatory cells are recruited, migrate and proliferate at the site of inflammation, large amounts of cytokines are released, with an up-regulation of glucose transporters (GLUT) (especially GLUT1 and GLUT3) and increased hexokinase (subtype A) activity, which result in enhanced glucose metabolism and FDG uptake in inflammatory cells (7). MET is a radiotracer whose uptake is regulated by transmembrane transport by the sodium-independent L-amino acid transporter (8). It reflects intracellular metabolic activity and is proportional to cell proliferation in neuro-oncological diseases (8). Its use is also rising in imaging inflammatory diseases, as inflammation is accompanied by proliferating cells that have elevated amino acid uptake, such as pro-inflammatory macrophages (8). Therefore, FDG- and MET-PET are imaging modalities that have the ability to evidence inflammation.

In systemic large vessel vasculitis, the usefulness of FDG-PET in detecting soft tissue involvement is well-known (9). In a recent study, FDG-PET had a sensitivity of 87% and a specificity of 84% in Takayasu arteritis, rising up to respectively 90 and 98% in giant cell arteritis (10). For medium and small vessels systemic vasculitis, several case series have shown that FDG-PET detect soft tissue inflammation earlier compared to other imaging modalities (11). Besides confirmation by pathology, definitive diagnosis of svPACNS requires the exclusion of mimickers, especially extra-CNS inflammation or brain tumors. One study reports an extra-CNS FDG-PET positive lesion in 90% of patients with small vessel ANCA positive vasculitis (12). Therefore, as illustrated by our two cases, a negative whole body FDG-PET is a useful indication in the management of this rare disease. Moreover, this sensitive whole-body imaging modality allows also the detection of extracerebral CNS involvement. This was the case in Patient 2 in whom spinal cord involvement was evident on the whole body FDG-PET performed at disease recurrence.

Very few studies or case reports have specifically searched if brain regions involved in svPACNS show a specific metabolic pattern on brain PET imaging. One case report about a biopsy proven svPACNS showed a negative brain MET-PET (13). Nevertheless, it should be noted that the PET was performed several days after the initiation of steroids, which increases the risk of a false negative result. A second study has tested the value of [18F]DPA-714 TSPO, a tracer of microglial activation, in nine patients suspected of svPACNS including two biopsy-proven cases (14). Results showed an increased uptake of the tracer in these two biopsy-proven patients. They suggest that it would be interesting to carry follow-up PET because of the reduced lesional uptake after anti-inflammatory treatment. PET imaging may thus facilitate the treatment monitoring of PACNS. Another case report described a biopsy-proven case of svPACNS in whom PET with an amino-acid tracer (O-(2-[F]fluoroethyl)-L-tyrosine) was positive (15). We could evidence FDG (Patient 1) and MET (Patient 2) increased uptake in the brain regions involved in small vessels inflammation, suggesting that brain PET imaging is an interesting diagnostic tool in svPACNS.

No studies have searched to what extent there is a match between brain MRI signal abnormalities and areas of increased PET tracer uptake in svPACNS. Interestingly, in our two biopsy-proven svPACNS, we found an increased FDG uptake for Patient 1 and an increased MET uptake for Patient 2 in brain regions with but also without brain MRI abnormalities [see Figures 1A,B and Figure 1A in (6)]. This mismatch between brain MRI and PET is also evidenced in brain tumors as the metabolically active tumor burden extends considerably beyond the volume of MRI (5). As already mentioned, brain biopsy is the gold standard to confirm the diagnosis of svPACNS in order to evidence the perivascular lymphocytic infiltration. To ensure high diagnostic yield, this procedure needs to be “en bloc” incisional biopsy prior to electrocautery, with a volume of at least 1 cm^3^, containing leptomeninges, cortical gray matter and subcortical white matter (4). The diagnostic value of brain biopsy is also influenced by an adequate targeting of regions where brain inflammation is at the highest (4). Therefore, since our two cases demonstrate a mismatch between brain MRI and PET abnormalities, we suggest that using both imaging modalities to target brain biopsy, just as in brain tumors, might be an interesting strategy to increase the diagnostic yield of svPACNS. The strength of PET imaging in svPACNS lies in its association to brain biopsy. Limitations of PET include diagnosis delay due to limited availability, and the possible influence of previous immunosuppressive therapy on the findings, especially glucocorticoids that are often administrated in the early course of the disease. PET and brain biopsy should ideally be performed before initiating immunosuppressive treatment.

## Conclusion

These two biopsy-proven cases of svPACNS demonstrate that both brain FDG- and MET-PET can detect a high metabolic activity in svPACNS that extends beyond brain MRI abnormalities. In addition, whole body FDG-PET is useful to exclude extra-CNS involvement. Combined MRI and PET should be systematically performed when suspecting svPACNS, preferentially before initiating immunosuppressive treatment, to adequately guide brain biopsy. If further work confirms these findings, it will aid the diagnosis of this challenging disease. As the main limitation of this study is the small number of patients, systematic studies are needed to confirm this on a larger scale.

## Data Availability Statement

The original contributions presented in the study are included in the article/supplementary material, further inquiries can be directed to the corresponding author/s.

## Author Contributions

MB wrote the article. PD participated in the literature review and writing. AM contributed to the diagnosis. OD contributed to the diagnosis by performing the brain biopsy. LL and IS contributed to the diagnosis by interpreting the anatomopathological samples. SG and XD contributed to the diagnosis by analyzing the imaging results and brought their great expertise in the field. AA contributed to the diagnosis, participated in the writing, and performed several proofreadings to improve the content. All authors contributed to the article and approved the submitted version.

## Conflict of Interest

The authors declare that the research was conducted in the absence of any commercial or financial relationships that could be construed as a potential conflict of interest.

## Publisher's Note

All claims expressed in this article are solely those of the authors and do not necessarily represent those of their affiliated organizations, or those of the publisher, the editors and the reviewers. Any product that may be evaluated in this article, or claim that may be made by its manufacturer, is not guaranteed or endorsed by the publisher.

## Abbreviations

ANA: anti-nuclear antibody
ANCA: antineutrophil cytoplasmic antibody
CNS: central nervous system
CSF: cerebrospinal fluid
EEG: electroencephalogram
FDG: [^18^F]–fluorodeoxyglucose
FLAIR: fluid attenuated inversion recovery
GLUT: glucose transporters
MET: [^11^C]-methionine
MOG: anti-myelin oligodendrocyte glycoprotein
MRI: magnetic resonance imaging
PACNS: primary angiitis of the central nervous system
PET: positron emission tomography
RF: rheumatoid factor
svPACNS: small vessel primary angiitis of the central nervous system.
